# Impact of an Intensive Care Information System on the Length of Stay of Surgical Intensive Care Unit Patients: Observational Study

**DOI:** 10.2196/14501

**Published:** 2019-09-04

**Authors:** Camille Havel, Jean Selim, Emmanuel Besnier, Philippe Gouin, Benoit Veber, Thomas Clavier

**Affiliations:** 1 Department of Anesthesiology and Critical Care Rouen University Hospital Rouen France; 2 Normandie Univ, UNIROUEN, INSERM U1096 Rouen France

**Keywords:** intensive care unit, length of stay, software, critically ill patient

## Abstract

**Background:**

The implementation of computerized monitoring and prescription systems in intensive care has proven to be reliable in reducing the rate of medical error and increasing patient care time. They also showed a benefit in reducing the length of stay in the intensive care unit (ICU). However, this benefit has been poorly studied, with conflicting results.

**Objective:**

This study aimed to show the impact of computerization on the length of stay in ICUs.

**Methods:**

This was a before-after retrospective observational study. All patients admitted in the surgical ICU at the Rouen University Hospital were included, from June 1, 2015, to June 1, 2016, for the before period and from August 1, 2016, to August 1, 2017, for the after period. The data were extracted from the hospitalization report and included the following: epidemiological data (age, sex, weight, height, and body mass index), reason for ICU admission, severity score at admission, length of stay and mortality in ICU, mortality in hospital, use of life support during the stay, and ICU readmission during the same hospital stay. The consumption of antibiotics, biological analyses, and the number of chest x-rays during the stay were also analyzed.

**Results:**

A total of 1600 patients were included: 839 in the before period and 761 in the after period. Only the severity score Simplified Acute Physiology Score II was significantly higher in the postcomputerization period (38 [SD 20] vs 40 [SD 21]; *P*<.05). There was no significant difference in terms of length of stay in ICU, mortality, or readmission during the stay. There was a significant increase in the volume of prescribed biological analyses (5416 [5192-5956] biological exams prescribed in the period before Intellispace Critical Care and Anesthesia [ICCA] vs 6374 [6013-6986] biological exams prescribed in the period after ICCA; *P*=.002), with an increase in the total cost of biological analyses, to the detriment of hematological and biochemical blood tests. There was also a trend toward reduction in the average number of chest x-rays, but this was not significant (0.55 [SD 0.39] chest x-rays per day per patient before computerization vs 0.51 [SD 0.37] chest x-rays per day per patient after computerization; *P*=.05). On the other hand, there was a decrease in antibiotic prescribing in terms of cost per patient after the implementation of computerization (€149.50 [$164 USD] per patient before computerization vs €105.40 [$155 USD] per patient after computerization).

**Conclusions:**

Implementation of an intensive care information system at the Rouen University Hospital in June 2016 did not have an impact on reducing the length of stay.

## Introduction

### Background

Medical and paramedical staff working in intensive care units (ICUs) need to collect and process a lot of data regarding patient care (eg, vital parameters, drug prescriptions and biological analyses, and changes in multiple daily prescriptions). The management of these data represents a time that is not directly devoted to patient care, and this can lead to errors in the prescription or delivery of treatments or may delay care.

To increase both the time dedicated to patients and the quality of care, ICU has been equipped with clinical information system (intensive care information system, ICIS) [1-5] over the past 20 years. These clinical information systems have demonstrated their effectiveness in reducing medical errors and improving patient safety [6]. Indeed, many studies have focused on showing the effect of computerization on the reduction of medical errors, whether in ward or in ICU. They all showed a reduction in the rate of medical errors by up to 80% and a better detection of errors before they were committed [7-10]. In addition, it has been shown that errors made were less serious and had fewer serious side effects [10]. It has also been shown that clinical information systems lead to an increase in the amount of time dedicated to patients’ care through a reduction in the time spent consulting and collecting the medical data [11,12].

The impact of information systems on the departments’ organization and the length of stay has been less studied. It is known that adverse drug events in hospitalized patients increase the length of stay and induce extra cost [13]. Thus, given the beneficial impact of information systems on medical and treatment delivery errors, we could have expected a beneficial impact of these systems on the length of hospital stay. However, the impact of computerization on the length of stay in ICU has been poorly studied, with conflicting data. In 2014, Levesque et al found a reduction in the length of stay in ICU of approximately 2 days following ICIS implementation [14], but this study only concerned critically ill patients from a specialized ICU. In addition, a study conducted on all the services of a university hospital before and after computerization found an overall reduction in the length of stay in the medical and surgical services but an increase in the length of stay in most ICUs [15]. Finally, a third study did not find any impact of ICIS on length of stay in ICU [16].

Intellispace Critical Care and Anesthesia (ICCA) is a recent ICIS developed by Philips Healthcare (Amsterdam, Holland). It allows a precise prescription of treatments (administration route, doses, administration times, and duration) as well as the automatic collection of a patient’s vital constants from bedside monitors and ventilators. It also contains the medical file and gathers the information essential to the patient’s care. The literature on the impact of ICIS on the length of stay is controversial, and there is no report on the impact of ICCA software (Philipps) on the quality of care for critically ill patients.

### Objective

The objective of this study was to assess the potential impact of ICCA on the length of stay of patients admitted in ICU.

## Methods

### Study Design

This was a retrospective before-after observational study conducted in an adult surgical ICU of a tertiary care hospital. This study received a favorable opinion from the Ethics Committee for Non-Interventional Research of the Rouen University Hospital (nE2018-55). We compared 2 periods: 1 period before the implementation of ICCA (from June 1, 2015, to June 1, 2016) and the other after its implementation (from August 1, 2016, to August 1, 2017). We excluded the 2 months following implementation of ICCA from the analysis to avoid the bias of software discovery by medical and paramedical staff, as previously described [14].

During these 2 periods, all adult patients hospitalized in ICU were included. For each patient, we collected epidemiological data (age, sex, weight, height, and body mass index), reason for ICU admission (trauma, vascular surgery, visceral surgery, thoracic surgery, other major surgery, medical or surgical sepsis, posttraumatic and postoperative hemorrhagic shock, and other reasons), severity score at admission (Simplified Acute Physiology Score II [SAPSII]), mortality and length of stay in ICU, readmission in ICU, and the use of life support during the stay (catecholamine, mechanical ventilation, and dialysis). Concerning the readmission rate, we have considered any readmission in ICU during the same hospital stay, whether or not it was for the same reason; only the first stay was analyzed. The data collection was based on the patient’s hospitalization report available on the hospital’s software.

During these periods, we also collected the number of chest x-rays prescribed during the stay; the consumption of biological analyses (bacteriological, biochemical, hematological, pharmacological, and virological analyses), collected in terms of total cost and prescribed volume month by month; and antibiotic consumption (in terms of total cost and delivery volume from the hospital’s central pharmacy). The consumption of complementary exams (chest x-rays and biological analyses) as well as antibiotic consumption only concerned the ICU stay included in the analysis if the patient had been readmitted in ICU. Concerning biological analyses and antibiotic consumption, we have obtained these data from persons not working in our department; their data extracting method is unknown to us. It should be noted that during the 2 years of inclusion of our study, the recommendations of the French Society of Anesthesia and Resuscitation regarding the correct prescribing practices for complementary exams were not modified, nor were the department’s internal recommendations.

The primary objective of this study was to compare the average length of stay in surgical ICU before and after implementation of ICCA. The secondary objectives were to analyze mortality, readmission in ICU during the same stay, as well as the consumption of additional examinations and antibiotic therapy (in terms of total quantity, total cost, and cost per patient over the period studied) as indirect indicators of the quality of medical prescriptions.

### Intellispace Critical Care and Anesthesia

The computerization of our ICU took place in June 2016. It was based on the ICCA software implementation. This computer support includes the patient’s medical file, computerized medical prescriptions, as well as the patient’s monitoring data. Computerized prescription is done using drop-down menus, classified by category (continuous, discontinuous drugs, additional tests, and biological analyses). All treatments referenced at the hospital central pharmacy are integrated into the software, and there are preconfigured prescriptions with proposed treatment regimens for some commonly used treatments (Multimedia Appendix 1). Similarly, most of the additional tests are available as drop-down menus with ticked items, such as the additional biological tests available at the hospital (Multimedia Appendix 1).

The patient monitoring signs include the different vital constants, ventilation parameters, various drainage systems, and so on, which are necessary for monitoring critically ill patients (Multimedia Appendix 2). Some data are extracted from the ICU devices (data from scopes, ventilators, and electric syringes), allowing real-time monitoring of the patient’s progress as well as the various therapies administered. There is also the possibility of manually entering the data, when they are not automatically collected (eg, diuresis), when the automatic data are incorrect, or when the feedback is interrupted (eg, connection broken between the ICU devices and the software).

### Statistical Analysis

On the basis of the publication of Levesque et al and our estimated mean length of stay in ICU (7 days), we assumed that a difference of 2 days (with a SD of 12 days) between the 2 groups would be clinically significant [14]. On the basis of these results, assuming that the SD was the same between populations and using a power of 0.90 with a statistical significance level of .05, we estimated that a minimum of 757 patients should be analyzed in each group, representing an inclusion duration of 1 year for each period.

Patients from both periods were compared by statistical analyses based on comparisons of means (Student test and Mann-Whitney test) and contingency analyses (chi-square). The statistical analyses were performed using the GraphPad Prism 7 software (GraphPad Software, USA). Quantitative variables are presented as mean (SD) if the distribution respected the normal law or median (interquartile range); if not, qualitative variables are expressed as absolute number and percentage.

## Results

### Epidemiological Data

Over the study periods, 1600 patients were included: 839 during the before period and 761 during the after period. The 2 groups of patients were comparable in terms of age and sex (Table 1). We observed a significant difference concerning the reason for ICU admission with more bleeding shock after computerization (6.4% (54/839) before ICCA vs 9.3% (71/761) after ICCA; *P*=.04) and less other major surgery after computerization (10.9% (91/761) before ICCA vs 6.6% (50/761) after ICCA; *P*=.003). SAPSII at ICU admission was lower in the before group (SAPSII: 38 [SD 20] vs 40 [SD 21] after computerization; *P*<.05; Table 1).

**Table 1:** Epidemiological data.

Data analyzed		Before ICCA^a^	After ICCA	*P* value
Age (years), mean (SD)		57.8 (17.8)	56.7 (18.8)	.22
**Sex, n (%)**				
	Male	537 (64.0)	505 (66.4)	.34
	Female	302 (36.0)	257 (33.6)	.34
Body mass index, mean (SD)		26.6 (6.3)	26.3 (6)	.38
Simplified Acute Physiology Score II, mean (SD)		38 (20)	40 (21)	.02^b^
**Reason for hospitalization, n (%)**				
	Sepsis^c^	114 (13.6)	106 (13.9)	.88
	Trauma	132 (15.8)	145 (19.0)	.09
	Vascular surgery	64 (7.6)	57 (7.5)	.92
	Gastrointestinal surgery	97 (11.6)	78 (10.2)	.42
	Bleeding shock^d^	54 (6.4)	71 (9.3)	.04^b^
	Thoracic surgery	41 (4.9)	36 (4.7)	.91
	Other major surgery	91 (10.9)	50 (6.6)	.003^b^
	Other cause	244 (29.1)	218 (28.7)	.83

^a^ICCA: Intellispace Critical Care and Anesthesia.

^b^Significant results.

^c^All medical or surgical sepsis.

^d^Traumatic or postoperative bleeding shock.

### Length of Stay and Mortality

All the ICU stay characteristics of the 2 groups are summarized in Table 2. There was no difference in the length of stay in ICU between the groups (7.0 (SD 9.3) days before computerization vs 7.4 (SD 9.9) days after computerization; *P*=.37). Mortality in the 2 groups did not differ significantly, nor did the readmission rate during the same stay and the use of different life supports (Table 2).

**Table 2:** Intensive care unit stay characteristics of the 2 groups.

Data analyzed		Before ICCA^a^	After ICCA	*P* value
Length of stay (days), mean (SD)		7.0 (9.3)	7.4 (9.9)	.37
**Mortality, n (%)**				
	In intensive care unit	139 (16.6)	128 (16.8)	.89
	In hospital	181 (21.6)	171 (22.5)	.67
Re-admission during the same stay, n (%)		59 (7.0)	60 (7.9)	.57
**Mechanical ventilation, n (%)**		590 (70.3)	554 (72.8)	.29
	Length of time (days), mean (SD)	6.1 (9.4)	6.9 (10.3)	.17
**Catecholamine, n (%)**		327 (39)	326 (42.8)	.13
	Length of time (days), mean (SD)	3.4 (5.3)	3.7 (7.0)	.41
**Dialysis therapy, n (%)**		92 (10.9)	98 (12.9)	.25
	Length of time (days), mean (SD)	7.2 (9.4)	7.6 (11.3)	.79
All 3 life supports (ventilation, vasopressor, and dialysis), n (%)		56 (6.7)	67 (8.8)	.11

^a^ICCA: Intellispace Critical Care and Anesthesia.

### Indirects Markers of Medical Prescriptions

Concerning the markers of medical prescriptions, we noted a significant increase in the volume of prescribed biological analyses, with an increase in the total cost of biological analyses, with a significant increase of hematological and biochemical blood tests. The monthly consumption and cost of biological analyses are summarized in Table 3.

There was no significant difference in the number of chest x-rays prescribed during the 2 study periods (0.55 [SD 0.39] chest x-rays per day of hospitalization per patient before the introduction of ICCA and 0.51 [SD 0.37] chest x-rays per day of hospitalization per patient after the introduction of ICCA; *P*=.05).

There was a decrease in the cost of antibiotic prescription per patient after the introduction of ICCA (€149.50 [$164 USD] per patient before computerization vs €105.40 [$116 USD] per patient after computerization), but the quantity of delivery antibiotics was globally stable in the 2 periods. In addition, in view of the change of markets during the 2 study periods (eg, with a wider use of generics), we decided to repeat the comparison while keeping the cost of antibiotics constant between the 2 periods. We then found a cost per patient of €149.50 ($164 USD) per patient before computerization vs €177.20 ($194 USD) per patient after computerization.

**Table 3:** Monthly consumption and cost of biological analyses.

Consumption of biological test	Before ICCA^a^, median (IQR^b^)	After ICCA, median (IQR)	*P* value
Total volume of biological analyses (number of acts)	5416 (5192-5956)	6374 (6013-6986)	.002^c^
Total cost of biological analyses (€)	28,503 (25,531-29,270)	32,530 (30,222-35,973)	.01^c^
Volume of hematological blood test (number of acts)	1032 (955-1094)	1182 (1012-1215)	.04^c^
Volume biochemical blood test (number of acts)	1628 (1383-1652)	1899 (1675-2062)	.02^c^
Volume of procalcitonin and troponin (number of acts)	85 (80-104)	94 (63-123)	.70
Volume of antibiotic dosage (number of acts)	79 (71-87)	81 (59-96)	.90

^a^ICCA: Intellispace Critical Care and Anesthesia.

^b^IQR: interquartile range.

^c^Significant results.

## Discussion

### Principal Findings

The implementation of ICIS with ICCA in June 2016 was not associated with a significant modification in the length of stay in our surgical ICU for critically ill patients. These results are the same as those of 2 studies, which reported that after implementation of ICIS in their ICU, the length of stay and morbidity and mortality of hospitalized patients were not affected by the change in prescribing patterns [16,17]. The lack of impact of computerization contradicts the data of Levesque et al who found a reduction in the length of stay of around 2 days after implementation of ICIS, whereas Lyons et al showed an increase in the length of stay in ICU after computerization [14,15]. However, the comparison of these studies remains difficult because each one tested a different software and there is no evidence that these systems are equivalent. To our knowledge, there is no study comparing different types of ICU ICIS software. Given evident logistical constraints, it seems difficult to envisage a randomized study comparing several ICIS software in the same ICU.

We showed that the postcomputerization population was sicker that the precomputerization population, via a significant increase in the severity score SAPSII between the 2 populations. If this difference is statistically significant, we did not consider it clinically relevant. Indeed, the difference between the 2 groups was only 2 points in the severity score, with relatively high scores. The difference was, therefore, small compared with the high average severity scores of our cohorts. One hypothesis is that this may be due to an overall increase in the age of the ICU populations (not demonstrated in our study).

Regarding secondary parameters, very few studies are available. Several studies have evaluated the impact of computerization on the mortality of critically ill patients, particularly for pediatric patients, but they did not show any effect [16-20]. Only a study by Lyons et al showed a reduction in mortality after computerization (3 deaths avoided per 1000 hospitalizations; *P*<.001), which was limited to medical and surgical wards. On the other hand, in this study, the implementation of ICIS was associated with an increase in mortality in ICU [15].

Many studies have focused on demonstrating the safety of computerized surveillance and prescription systems [6-8]. They all showed a reduction in the rate of medication delivery errors and an increase in the rate of errors identified before they were committed [8-10]. However, there is no study looking for a qualitative change of medical prescriptions after the implementation of an ICIS in ICU. We did not analyze every individual medical prescription, and therefore, we did not have direct data concerning prescription errors or practices (eg, forgetting to repeat treatment when prescribing manually). Prescriptions were analyzed indirectly via the quantity of treatments or additional exams consumed. We selected 3 quality markers of medical prescriptions: the consumption of antibiotics (whose duration is fixed and unchanged regardless of the mode of prescription), the consumption of biological analyses (estimated by total cost), and the average number of chest x-rays during the stay. In our study, a reduction in the average cost of antibiotic therapy per patient after implementation of ICCA was observed, but there was no clinically significant difference in the amount of antibiotics delivered. The cost difference was due to a change in market with a large use of generic molecules (as the replacement between the 2 periods of the delivery of Zyvoxid by generic Linezolid) and a diversification of the drugs used (such as the use of fourth-generation cephalosporins and new glycopeptides to fight against multiresistant bacteria and to preserve carbapenems).

The implementation of ICCA was associated with a significant increase in the total cost and prescription of biological tests after computerization, whereas the service’s recommendations concerning the prescription of additional tests remained the same over these periods. We observed that the increase in the cost of biological tests did not correspond to the overprescription of some expensive biological tests (eg, troponin or procalcitonin or antibiotics dosages) but corresponded to an increase of biochemical and hematological blood tests prescribed. This over-prescription could have been facilitated by the ease of prescription through a drop-down menu (leading to the prescription of nonrecommended tests). Collin et al who analyzed the impact of the implementation of an information system for medical prescription in 4 general hospitals of the National Health Service in Great Britain observed similar results: the prescription of additional tests was almost quadrupled in patients hospitalized in conventional departments after computerization [21]. This increase of the volume of prescription of additional tests may be due to a change in practices. Indeed, we have the impression that younger generations of physicians used to have easy access to biological and radiological exams, and they seem more likely to rely on complementary exams rather than physical assessment to diagnose and treat patients. A major side effect of this trend is an increase in the cost of care, which is becoming increasingly central to the overall management of patients.

It has been shown that the computerization of the storage and retrieval system for radiological examinations leads to a reduction in the repetition of their prescription [21,22]. In our work, the introduction of ICCA had led to a substantial, but not significant, reduction in the average number of chest x-rays prescribed during the stay. This may be part of a trend toward a better follow-up of professional recommendations, which recommends not systematically prescribing a daily chest x-ray to any intubated patient [23].

Surveillance and prescription software have already shown a benefit in terms of the amount of care provided to patients by reducing the time spent consulting and collecting the data needed to manage them [24]. Saarinen et al found that computerization in ICU increased the time spent in patient care (81.1% of working time before computerization vs 86.6% after; *P*<.05) [11]. Similarly, in 2010, Ballermann et al showed a decrease in the time spent consulting the various care documents after setting up a computerized system in their department [12]. However, the efficiency of ICIS in terms of improving the quality of care remains debated. For example, Koppel et al showed that computerized prescription resulted in an increase of 22 types of medication errors, including double prescriptions without computer alerts, confusion of doses and pharmacy inventories, and a lack of overall vision of patient treatments through fragmented computer windows [25]. From indicators such as average length of stay, mortality, and indirect markers of prescription quality, our study suggests that the global quality of care has not been impacted by the implementation of an ICIS. The question that we are raising of whether computerization has a real impact on the prescription of complementary tests and antibiotic therapy could be specified in a future study detailing the individual quality of medical prescription.

Our study faces several strong limitations. First, only the length of stay of the first stay was analyzed. Therefore, each patient was included only once in the analysis. This is a bias in the comparison of the length of stay between the 2 study periods. However, as readmission rates are comparable between the 2 periods, we can consider that this balances the bias caused by the exclusion of successive stays of the same patient.

Furthermore, the quality of medical prescriptions was analyzed indirectly by collecting the quantity of additional biological and radiological tests prescribed as well as the quantity of antibiotics consumed during the 2 study periods. We did not collect and directly analyze prescriptions; hence, some errors such as duplicates, missed retreatments, or dose errors were not taken into account.

Moreover, this retrospective study did not allow us to gather the opinions of caregivers, nurses, and physicians on the computerization of the medical monitoring and prescription system. It has been shown that medical and paramedical caregivers have a positive experience with computerization, but we do not know what the impact of ICCA on our team was [26,27]. Finally, we excluded from our analysis the 2 months following the computerization of medical prescriptions. This may not be sufficient to judge a truly efficient use of the software for both medical and paramedical staff.

### Conclusions

On the basis of the indicators collected, the implementation ICCA in our surgical ICU, despite the significant changes it brings about daily practices, did not seem to impact the length of stay in ICU. The real impact of computerization on length of stay and morbidity and mortality in ICU remains controversial. A more detailed approach of medical prescription should better assess the possible benefits of computerized prescription in the specific population of critically ill patients.

## Appendix

Multimedia Appendix 1 Computerized medical prescriptions in ICCA; review of daily prescriptions, listed by route of administration and alphabetical order; example of a new prescription, with drop-down and preconfigured prescription menu, precision of route of administration, dosage, frequency and duration.

Multimedia Appendix 2 ICCA monitoring sheet, showing the different monitored vital parameters, classified by organs (hemodynamics, ventilation...) and function (drainage, analgesia...). The data are automatically validated every 30 minutes, with the possibility of correcting outliers or manually entering data remaining.

## Abbreviations

ICCA: Intellispace Critical Care and Anesthesia
ICIS: intensive care information system
ICU: intensive care unit
SAPSII: Simplified Acute Physiology Score II

## References

[1] Langenberg CJ (1996). Implementation of an electronic patient data management system (PDMS) on an intensive care unit (ICU). Int J Biomed Comput.

[2] Varon J, Marik PE (2002). Clinical information systems and the electronic medical record in the intensive care unit. Curr Opin Crit Care.

[3] Egan M (2006). Clinical dashboards: impact on workflow, care quality, and patient safety. Crit Care Nurs Q.

[4] Pennisi MA, Campioni P, Frassanito L, Maviglia R, Mignani V, di Nunno S, Costa R (2001). [Diagnostic imaging and patient database managing systems: the integration of digital information in the experience of an intensive care center]. Radiol Med.

[5] Bria WF, Shabot MM (2005). The electronic medical record, safety, and critical care. Crit Care Clin.

[6] Khammarnia M, Sharifian R, Zand F, Barati O, Keshtkaran A, Sabetian G, Shahrokh N, Setoodezadeh F (2017). The impact of computerized physician order entry on prescription orders: a quasi-experimental study in Iran. Med J Islam Repub Iran.

[7] Nuckols TK, Smith-Spangler C, Morton SC, Asch SM, Patel VM, Anderson LJ, Deichsel EL, Shekelle PG (2014). The effectiveness of computerized order entry at reducing preventable adverse drug events and medication errors in hospital settings: a systematic review and meta-analysis. Syst Rev.

[8] Bates DW, Leape LL, Cullen DJ, Laird N, Petersen LA, Teich JM, Burdick E, Hickey M, Kleefield S, Shea B, Vliet MV, Seger DL (1998). Effect of computerized physician order entry and a team intervention on prevention of serious medication errors. J Am Med Assoc.

[9] van Rosse F, Maat B, Rademaker CM, van Vught AJ, Egberts AC, Bollen CW (2009). The effect of computerized physician order entry on medication prescription errors and clinical outcome in pediatric and intensive care: a systematic review. Pediatrics.

[10] Colpaert K, Claus B, Somers A, Vandewoude K, Robays H, Decruyenaere J (2006). Impact of computerized physician order entry on medication prescription errors in the intensive care unit: a controlled cross-sectional trial. Crit Care.

[11] Saarinen K, Aho M (2005). Does the implementation of a clinical information system decrease the time intensive care nurses spend on documentation of care?. Acta Anaesthesiol Scand.

[12] Ballermann MA, Shaw NT, Arbeau KJ, Mayes DC, Gibney RT (2010). Impact of a critical care clinical information system on interruption rates during intensive care nurse and physician documentation tasks. Stud Health Technol Inform.

[13] Classen DC, Pestotnik SL, Evans RS, Lloyd JF, Burke JP (1997). Adverse drug events in hospitalized patients. Excess length of stay, extra costs, and attributable mortality. J Am Med Assoc.

[14] Levesque E, Hoti E, Azoulay D, Ichai P, Samuel D, Saliba F (2015). The implementation of an intensive care information system allows shortening the ICU length of stay. J Clin Monit Comput.

[15] Lyons AM, Sward KA, Deshmukh VG, Pett MA, Donaldson GW, Turnbull J (2017). Impact of computerized provider order entry (CPOE) on length of stay and mortality. J Am Med Inform Assoc.

[16] Al-Dorzi HM, Tamim HM, Cherfan A, Hassan MA, Taher S, Arabi YM (2011). Impact of computerized physician order entry (CPOE) system on the outcome of critically ill adult patients: a before-after study. BMC Med Inform Decis Mak.

[17] Wong DC, Knight J, Birks J, Tarassenko L, Watkinson PJ (2018). Impact of electronic versus paper vital sign observations on length of stay in trauma patients: stepped-wedge, cluster randomized controlled trial. JMIR Med Inform.

[18] Brunette DD, Tersteeg J, Brown N, Johnson V, Dunlop S, Karambay J, Miner J (2013). Implementation of computerized physician order entry for critical patients in an academic emergency department is not associated with a change in mortality rate. West J Emerg Med.

[19] del Beccaro MA, Jeffries HE, Eisenberg MA, Harry ED (2006). Computerized provider order entry implementation: no association with increased mortality rates in an intensive care unit. Pediatrics.

[20] Keene A, Ashton L, Shure D, Napoleone D, Katyal C, Bellin E (2007). Mortality before and after initiation of a computerized physician order entry system in a critically ill pediatric population. Pediatr Crit Care Med.

[21] Collin S, Reeves BC, Hendy J, Fulop N, Hutchings A, Priedane E (2008). Implementation of computerised physician order entry (CPOE) and picture archiving and communication systems (PACS) in the NHS: quantitative before and after study. Br Med J.

[22] Vecellio E, Georgiou A (2016). Integrating the radiology information system with computerised provider order entry: the impact on repeat medical imaging investigations. Stud Health Technol Inform.

[23] (2017). French Society of Anesthesia and Resuscitation.

[24] Wong D, Bonnici T, Knight J, Gerry S, Turton J, Watkinson P (2017). A ward-based time study of paper and electronic documentation for recording vital sign observations. J Am Med Inform Assoc.

[25] Koppel R, Metlay JP, Cohen A, Abaluck B, Localio AR, Kimmel SE, Strom BL (2005). Role of computerized physician order entry systems in facilitating medication errors. J Am Med Assoc.

[26] Ayatollahi H, Roozbehi M, Haghani H (2015). Physicians' and nurses' opinions about the impact of a computerized provider order entry system on their workflow. Perspect Health Inf Manag.

[27] Yusof MM (2015). A case study evaluation of a critical care information system adoption using the socio-technical and fit approach. Int J Med Inform.

