# Supplementary material for: Impact of an Intensive Care Information System on the Length of Stay of Surgical Intensive Care Unit Patients: Observational Study
Source: JMIR Perioper Med. 2019 Sep 4;2(2):e14501. doi: 10.2196/14501 (PMC7709852; doi:10.2196/14501)
Supplement: Multimedia Appendix 1 [file periop_v2i2e14501_app1.pdf]

Dossier Administratif | Dossier Médical | Dossier de Soins | Feuilles de surveillances | Prescriptions | Plan de soins | Scores et classifications | Plan de Soins | Tableaux de bord | Résultats labo | Documents externes

- P2 Vue / catégories pharmacothér
- P3 Nouvelles Prescriptions
- **Revue des prescriptions**

- Actes techniques
- Isolement & Surveillances
- Ventilation, Oxygénothér...
- Suppléances d'organes
- Médicaments
- Perf & Nutrition artificielle
- Régime alimentaire
- Analyses labo
- Radio, Endoscopie, Neur...
- Contention, Position, Réa...
- Avis spé, Consignes & So...
- Soins du rôle propre IDE

Allergies: - Aucune allergie connue

Taille (cm): 178 • IMC (habituel): 30,6 • Poids (habituel): 97 • S.C.: 2,15 • P1: • P2: • P3: • P4: • P5:

Revue des prescriptions : Médicaments

[toutes les 24 h] Prescription médicale

	[redacted] 00:00 - 23:59	[redacted] 00:00 - 23:59	[redacted] 00:00 - 23:59	[redacted] 00:00 - 23:59	[redacted] 00:00 - 23:59	[redacted] 00:00 - 23:59	[redacted] 00
IV ACIDE ASCORBIQUE (vitamine C)<br>1,5 g Toutes les 8 h<br>[redacted]	☑ 1,5 g IV 50 mL @ 03:00<br>☑ 1,5 g IV 50 mL @ 11:00<br>☑ 1,5 g IV 50 mL @ 19:00	☑ 1,5 g IV 20 mL @ 03:00<br>☐ Attente @ 11:00<br>☐ Attente @ 19:00	☐ Attente @ 03:00				
IV COLISTIMETHATE SODIQUE (colimycine)<br>3 M UI Toutes les 8 h<br>Instructions : Ce médicament est administré par voie intraveineuse en perfusion lente sur 60 minutes. Après reconstitution, la solution doit être diluée, habituellement avec 50 mL d'une solution de chlorure de [illegible]<br>[redacted]	☑ 3 M UI IV 50 mL @ 07:00<br>☑ 3 M UI IV 50 mL @ 15:00<br>☑ 3 M UI IV 50 mL @ 23:00	☑ 3 M UI IV 50 mL @ 07:00<br>☐ Attente @ 15:00<br>☐ Attente @ 23:00	☐ Attente @ 07:00				
IV METRONIDAZOLE (flagyl)<br>500 mg Toutes les 8 h<br>[redacted]	☑ 500 mg IV 100 mL @ 00:00<br>☑ 500 mg IV 100 mL @ 08:00<br>☑ 500 mg IV 100 mL @ 16:00	☑ 500 mg IV 100 mL @ 00:00<br>☑ 500 mg IV 100 mL @ 08:00<br>☐ Attente @ 16:00	☐ Attente @ 00:00				
IV OFLOXACINE (oflocet)<br>400 mg Toutes les 8 h<br>[redacted] lon GPR	☑ 400 mg IV 80 mL @ 03:00<br>☑ 400 mg IV 50 mL @ 11:00<br>☑ 400 mg IV 80 mL @ 19:00	☑ 400 mg IV 80 mL @ 03:00<br>☐ Attente @ 11:00<br>☐ Attente @ 19:00	☐ Attente @ 03:00				
IV PANTOPRAZOLE SEL DE NA<br>40 mg Toutes les 12 h<br>[redacted]	☑ 40 mg IV 50 mL @ 20:00	☑ 40 mg IV 50 mL @ 08:00<br>☐ Attente @ 20:00					
IV PARACETAMOL<br>1 g Toutes les 6 h si besoin<br>[redacted] 5°C							
IV PHYTOMENADIONE (vitamine k1)		☐ Attente @ 15:00					

Entrer nouvelles prescriptions

Allergies: - Aucune allergie connue

Taille (cm): 178 • IMC (habituel): 30,6 • Poids (habituel): 97 • S.C.: 2,15 • P1: • P2: • P3: • P4: • P5:

Mode: Prescription médicale

1. 5.a. Médicaments : Discontinus (IV, PO, etc...)
2. TEICOPLANINE
3. TÉICOPLANINE IV Toutes les 12 h

⊟ 5.a. Médicaments : Discontinus (IV, PO, etc...)

Médicament	*	TEICOPLANINE
Formule	*	TEICOPLANINE 400 mg ; voie...
Voie d'administration	*	IV
Dose par prise	*	mg
Dose max		
Solution de base		
Volume de dilution		50 mL
Fréquence	*	Toutes les 12 h
Début		30/10/2018 08:29
Horaire		30/10/2018 09:00
A passer en		30 min
Nombre de jours		
Fin		
Instructions prescription		
Info pour IDE		GEDI Ref 235<br>anti-infectieux fiche 172
Instructions de la phar...		
Fonction		Médecin
Livret		Oui

Ajouter autre | OK | Annuler
