# Supplementary material for: Impact of an Intensive Care Information System on the Length of Stay of Surgical Intensive Care Unit Patients: Observational Study
Source: JMIR Perioper Med. 2019 Sep 4;2(2):e14501. doi: 10.2196/14501 (PMC7709852; doi:10.2196/14501)
Supplement: Multimedia Appendix 2 [file periop_v2i2e14501_app2.pdf]

Dossier Administratif | Dossier Médical | Dossier de Soins | Feuilles de surveillances | Prescriptions | Plan de soins | Scores et classifications | Plan de Soins | Tableaux de bord | Résultats labo | Documents externes

- FS01 : Feuille de surveillanc
- FS02 : Soins de base et soins relat
- FS03 : Soins kinésithérapeutes
- FS04 : Suppléances d'organe
- FS05: Feuille de ventilation

- Vérifications à la Prise de...
- Actes et Evenements
- Surveillance de base
- Graphique paramètres vit...
- Graphique diurèse/neuro
- Hémodynamique
- Ventilation / Oxygénation
- Neurologie
- Drainages
- Bilan entrées/sorties
- Surveillance Analgésie
- Surveillances Spécifiques
- Conditionnement
- Précaut compl / isolement
- Surveillance (prescription)
- Médicaments discontinus
- Médicaments continus
- Remplissage & transfusion
- Perf/nutrition/apport hydr
- Labo
- ECG & autres examens c...
- Avis, consignes, sorties
- Contention position réad...
- Prescriptions infirmière
- IGS II & SOFA

FS01 : Feuille de surveillanc [redacted]

[Validation auto toutes 30 min]	15:00	16:00	17:00	18:00	19:00	20:00	21:00	22:00	23:00	30/10/2018 00:00	01:00	02:00	03:00	04:00	05:00	06:00	07:00	08:00
Poids (journalier)																		
Précautions		Contact			Contact			Contact			Contact			Contact			Contact	
FC	123	112	113	119	133	122	116	124	125	128	130	132	134	133	132	120	130	129
⊞ Pression artérielle non invasive																		
⊞ Pression artérielle invasive	129 /83 PAM : (102)	92 /60 PAM : (73)	102 /66 PAM : (81)	119 /78 PAM : (95)	142 /91 PAM : (113)	90 /59 PAM : (72)	75 /49 PAM : (61)	99 /63 PAM : (77)	119 /78 PAM : (94)	123 /78 PAM : (96)	144 /92 PAM : (112)	148 /97 PAM : (116)	126 /82 PAM : (99)	129 /80 PAM : (98)	105 /66 PAM : (81)	82 /50 PAM : (63)	117 /71 PAM : (88)	103 /63 PAM : (78)
PVC																		
Temp °(C)		37,2						37,5			36,8			38,2			38,1	
SpO2	93	93	98	98	97	93	91	94	97	97	97	96	93	93	99	94	93	94
Fréquence resp.	28	28	27	28	36	28	28	28	28	27	30	39	38	36	31	28	33	32
EtCO2																		
Hémocue		10 g/dl																
Glycémie capillaire		100						125			122			120			122	
⊞ Evaluation de la douleur		BPS : 3 EVS : 0			BPS : 3 EVS : 0			BPS : 3 EVS : 0			BPS : 3 EVS : 0			BPS : 3 EVS : 0			BPS : 3 EVS : 0	
Mode de ventilation		VI-VAC			VI-VAC	VI-VAC		VI-VAC			VI-VAC			VI-VAC			VI-VAC	
Conscience		Dort			Dort			Conscient			Dort			Dort			Dort	
RASS		Somnolent = -1			Somnolent = -1			Somnolent = -1			Diminution légère vigilance=-2			Diminution légère vigilance=-2			Diminution légère vigilance=-2	
Pertes, urine 24h	0 (+20)	0 (+20)	0 (+20)	0 (+20)	0 (+20)	0 (+20)	0 (+20)	0 (+20)	0 (+20)	0 (+20)	0 (+20)	0 (+20)	0 (+20)	0 (+20)	0 (+20)	0 (+20)	0 (+20)	
⊞ Drainage urinaire: Méat urétral Sonde vésicale		0			0			0			0			0			0	(20)
⊞ Drainage urinaire: Méat urétral Sonde vésicale		0			0			0			0			0			0	(20)
⊞ Stomie: Abdominal droit Colostomie					100			0			0						200	(300)
⊞ Drains thoraciques: Thorax gauche					30			0			10			10			10	(60)
⊞ Pertes Gastriques: Sonde Naso Gastrique								600										(600)
⊞ Drains: Drain abdominal droit					0			70			0			0			0	(70)
